# Supplementary material for: Somatic mutations in 3929 HPV positive cervical cells associated with infection outcome and HPV type
Source: Nat Commun. 2024 Sep 12;15:7895. doi: 10.1038/s41467-024-51713-y (PMC11393421; doi:10.1038/s41467-024-51713-y)
Supplement: Supplementary file 1 — Supplementary Information [file 41467_2024_51713_MOESM1_ESM.pdf]

Supplementary Information

Title: Somatic mutations in 3929 HPV positive cervical cells associated with infection outcome and HPV type

Maisa Pinheiro[1], Nicolas Wentzensen[1], Michael Dean[1,2], Meredith Yeager[1,2,3], Zigui Chen[4], Amulya Shastry[1,2], Joseph F. Boland[1,2], Sara Bass[1,2], Laurie Burdett[1,2], Thomas Lorey[5], Sambit Mishra[1,2], Philip E. Castle[1,6], Mark Schiffman[1], Robert D. Burk[7,8], Bin Zhu[1], Lisa Mirabello[1*]

Supplementary Methods

Mutation calling quality control

Amplicon panel sequence reads were mapped to hg19 using Torrent Mapping Alignment Program (TMAP; Thermo Fisher Scientific) with the following options: max-adapter-bases-for-soft-clipping = 25; end-repair = 15; min-al-len = 50. Only reads with a mapping quality of ≥4 were considered for variant calling. TVC v.5.0.3 (Thermo Fisher Scientific) was used for variant calling with the following parameters: snp_min_coverage = 100; snp_min_cov_each_strand = 4; snp_min_variant_score = 6; snp_min_allele_freq = 0.02; snp_strand_bias = 0.95; snp_strand_bias_pval = 0.01.

We used Acrometrix Oncology Hotspot Control DNA (AOH) to optimize the quality control filters for low variant allele fraction (VAF) mutations called by our targeted sequencing panel. This control panel consists of a mixture of genomic DNA, derived from the cell line DM24385 used to develop the Genome in a Bottle reference genome [1], and synthetic DNA, with known somatic mutations in cancers introduced at variant low allelic fractions. The AOH controls harbored 148 known mutations in eight of our 19 genes of interest (*TP53*, *STK11*, *PTEN*, *PIK3CA*, *KRAS*, *HRAS*, *FBXW7*, *ERBB2*), including 26 genomic variants (equivalent to germline variants), 37 variants with a target AF of 15-35% and 105 with a target AF of 5-15%. We sequenced this same control in triplicate, here called AOH-T1, AOH-T2 and AOH-T3, as part of 3 different experimental plates. We evaluated the expected and observed mutation VAFs and six additional VCF parameters generated by Torrent Variant Caller (TVC) related to variant quality and coverage including FILTER (passed TVC low-VAF parameters), QUAL (quality), FDP (flow evaluator read depth at the locus or total number of reads at the locus), FAO (flow

evaluator alternate allele observations or number of reads with alternate allele), STB (strand bias in variant relative to reference) and MLLD (mean log-likelihood delta per read). The threshold for each parameter was: FILTER = PASS, QUAL>=10, FDP>=100, FAO>=6, STB<0.9, MLLD>55. Out of 148 target mutations (137 SNVs, 3 insertions and 8 deletions), TVC called a total of 126 (85.1%) mutations with low-AF parameters, including 120 SNVs (88%), 2 insertions (50%) and 4 deletions (50%). For each independent replicate, 121 mutations were called in sample AOH-T1, 122 called in sample AOH-T2 and 122 called in sample AOH-T3. For samples AOH-T1, AOH-T2 and AOH-T3, respectively, 93.4%, 91.1% and 95.1% of mutations called with the low-AF parameters had concordant target and observed AF, while 8, 11 and 6 mutations were called below the target AF 5-15% (Figure S1).

HS mutation classification

First, for the CGI webtool we used the options "cancer type = cervix" and "reference genome = hg19". For Mutagene, we downloaded mutation annotation databases separately by each gene, using the "analyze gene tab", then selected the options "Cancer type = cervical squamous cell carcinoma" and "observed mutations = mutations in the selected cancer cohort" for mutations important for ICC, then selected the options "cancer type = Pan-Cancer" and "observed mutations = all genome wide studies in ICGC" for mutations important for other cancer types. For cBioPortal-TCGA-cervix, we downloaded the full cervical cancer mutation database and calculated the frequency of somatic mutation in that database by unique amino-acid (aa) change and by multiple mutations at the same aa position. For CHASMplus, we also downloaded the full database and focused on "common" mutations in "Pan-Cancer".

HPV genome sequencing and lineage/sublineage assignment

DNA from each woman in the study also had type-specific HPV16, HPV18, and/or HPV45 whole-genome sequencing [2-4]. DNA underwent library construction according to the manufacturer's recommendations using AmpliSeq Library Preparation kit 2.0-96LV (Thermo Fisher Scientifics) and custom oligonucleotide primers, designed by Life Tech in conjunction with our lab personnel, that amplify 46-48 amplicons covering 100% of the viral genomes. Amplification was performed using Phusion High-Fidelity DNA (Thermo Fisher Scientifics), with an error rate less than 1%. Individual libraries were quantified prior to sequencing using the

Kapa Biosystems Library Quantification Kit - IonTorrent/LightCycler480, and library concentration was determined using Agilent BioAnalyzer DNA High-Sensitivity LabChip (Agilent Technologies). Sequencing was performed on the Thermo Fisher Life Science Ion Torrent S5 GeneStudio systems (Thermo Fisher Scientifics). Raw sequence reads were quality assessed and trimmed, and then mapped to the HPV16, HPV18, or HPV45 reference complete genome sequences using Ion Torrent Suite software (Life Technologies, Thermo Fisher Scientifics). An in-house custom pipeline was used for variant calling and gene annotation using the Torrent Variant Caller v.5.0.3 and snpEff v.3.6c.

Each HPV sequence was assigned to an HPV type-specific lineage/sublineage based on the maximum likelihood phylogenetic tree topology constructed with RAxML MPI[5] and type-specific HPV16, HPV18 and HPV45 genome sequence FASTA files, including known lineage/sublineage reference sequences for that HPV type [3,4,6]. Each tree was bootstrapped 1,000 times, and sublineage assignments were assigned by proximity in the tree to known sublineage reference sequences. Each HPV16 sequence was classified as one of the following sublineages A1-A4, B, C, or D1-D4; HPV18 sequences were classified as sublineage A1-A5, or B; and, HPV45 sequences as sublineage A1, A2, B1, B2, or C1.

References

1 Zook, J. M. *et al.* Extensive sequencing of seven human genomes to characterize benchmark reference materials. *Sci Data* **3**, 160025 (2016). https://doi.org/10.1038/sdata.2016.25

2 Cullen, M. *et al.* Deep sequencing of HPV16 genomes: A new high-throughput tool for exploring the carcinogenicity and natural history of HPV16 infection. *Papillomavirus research* **1**, 3-11 (2015). https://doi.org/10.1016/j.pvr.2015.05.004

3 Pinheiro, M. *et al.* Phylogenomic Analysis of Human Papillomavirus Type 31 and Cervical Carcinogenesis: A Study of 2093 Viral Genomes. *Viruses* **13** (2021). https://doi.org/10.3390/v13101948

4 Pinheiro, M. *et al.* Association of HPV35 with cervical carcinogenesis among women of African ancestry: Evidence of viral-host interaction with implications for disease intervention. *Int J Cancer* **147**, 2677-2686 (2020). https://doi.org/10.1002/ijc.33033

5 Stamatakis, A. RAxML-VI-HPC: maximum likelihood-based phylogenetic analyses with thousands of taxa and mixed models. *Bioinformatics* **22**, 2688-2690 (2006). https://doi.org/10.1093/bioinformatics/btl446

6 Mirabello, L. *et al.* HPV16 Sublineage Associations With Histology-Specific Cancer Risk Using HPV Whole-Genome Sequences in 3200 Women. *JNCI: Journal of the National Cancer Institute* **108**, djw100-djw100 (2016). https://doi.org/10.1093/jnci/djw100

Supplementary Figures

Supplementary Figure 1. Target versus observed variant allele fraction (AF) of 137 known mutations called by the Torrent Variant Caller (TVC).

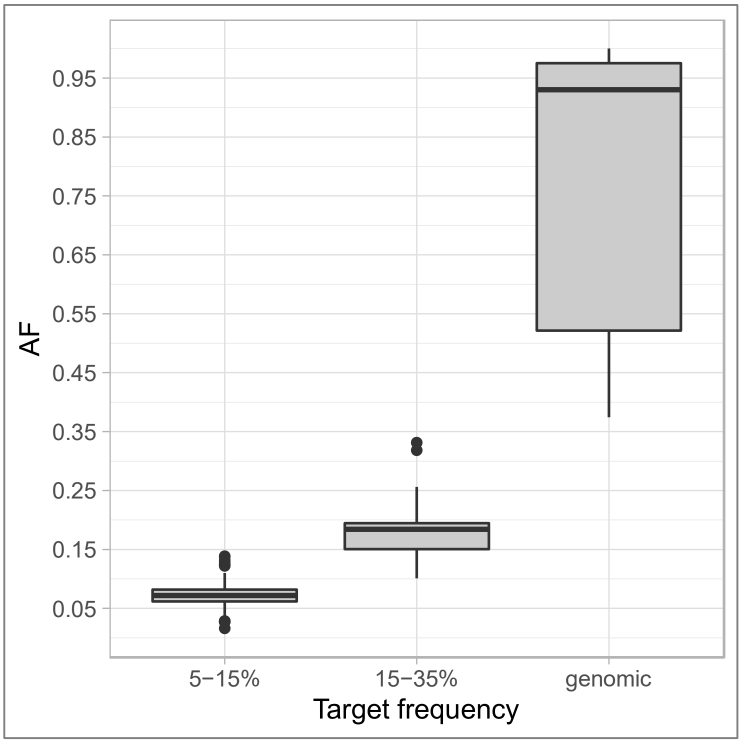

Supplementary Figure 2. Overall variant allele fraction (AF) of all mutations by TIER classification.

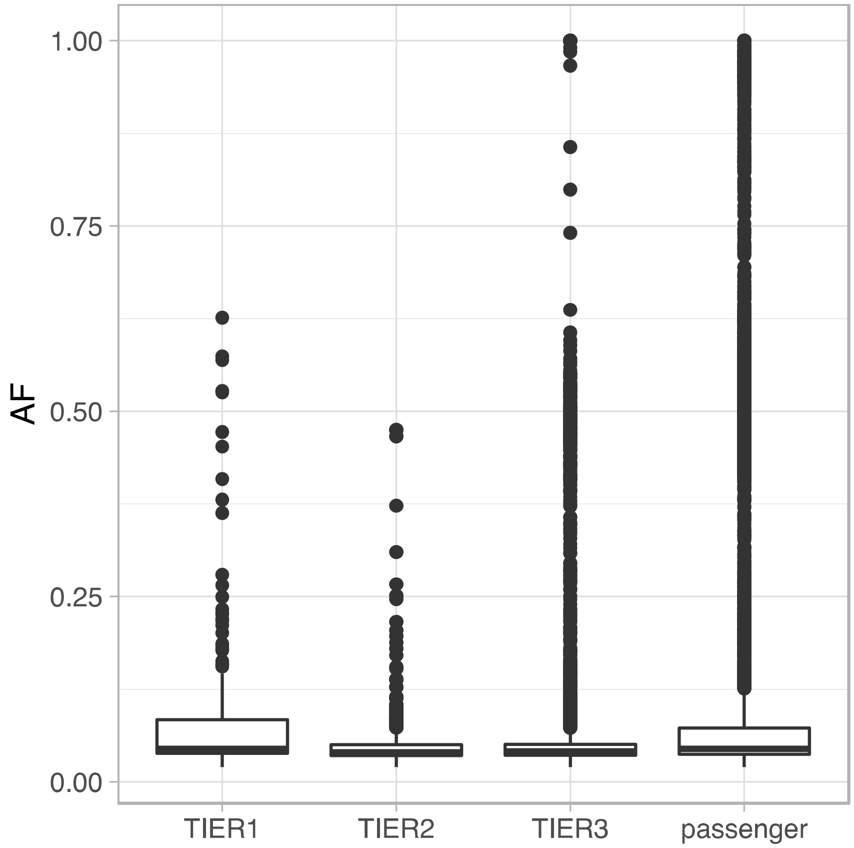

Mean VAF	0.093	0.049	0.063	0.126
Median VAF	0.045	0.040	0.041	0.045

Supplementary Figure 3. Odds ratios calculated using a generalized linear mixed-effects model for the association of hotspot mutations and the probability of being cancers instead of controls with HPV type lineages/sublineages as the random effect. The bars represent 95% confidence intervals (CIs) for the corresponding odds ratio estimates for each HPV lineage/sublineage. The horizontal red line represents the average odds ratio estimates across HPV lineages/sublineages.

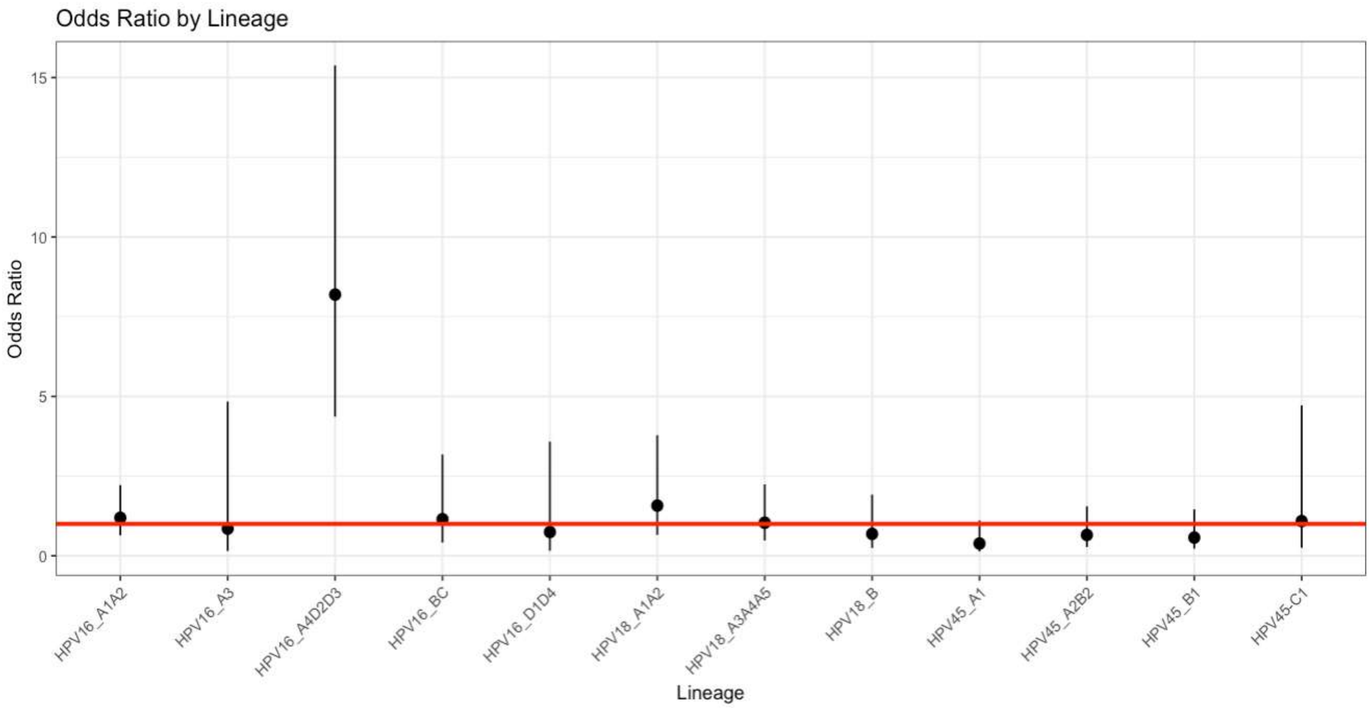

		HPV16 A1A2		HPV16 A3		HPV16 A4D2D3		HPV16 BC		HPV16 D1D4		HPV18 A1A2		HPV18 A3A4A5		HPV18 B		HPV45 A1		HPV45 A2B2		HPV45 B1		HPV45 C1	
		N	%	N	%	N	%	N	%	N	%	N	%	N	%	N	%	N	%	N	%	N	%	N	%
**CONTROL**	**No HS**	398*	86.3%	3	100.0%	43	55.1%	38	84.4%	6	100.0%	63	86.3%	135	88.2%	58	93.5%	84	95.5%	90	91.8%	77	93.9%	10	90.9%
	**≥1 TIER1 HS**	14	3.0%	0	0.0%	0	0.0%	2	4.4%	0	0.0%	1	1.4%	4	2.6%	1	1.6%	2	2.3%	3	3.1%	2	2.4%	0	0.0%
**CANCER**	**No HS**	34	7.4%	0	0.0%	27	34.6%	4	8.9%	0	0.0%	8	11.0%	9	5.9%	3	4.8%	1	1.1%	4	4.1%	3	3.7%	1	9.1%
	**≥1 TIER1 HS**	15	3.3%	0	0.0%	8	10.3%	1	2.2%	0	0.0%	1	1.4%	5	3.3%	0	0.0%	1	1.1%	1	1.0%	0	0.0%	0	0.0%

* Reference group. % = column percentage.

Supplementary Figure 4. Variant allele fraction (AF) of hotspot mutations by **a)** status in the single time-point analyses for TIER2 mutations, and by **b)** serial time-point analyses in non-cancer samples. P values were estimated using a two-sided Wilcoxon rank sum test with continuity correction. ns= not significant; * p value ≤0.05; *** p value ≤0.001.

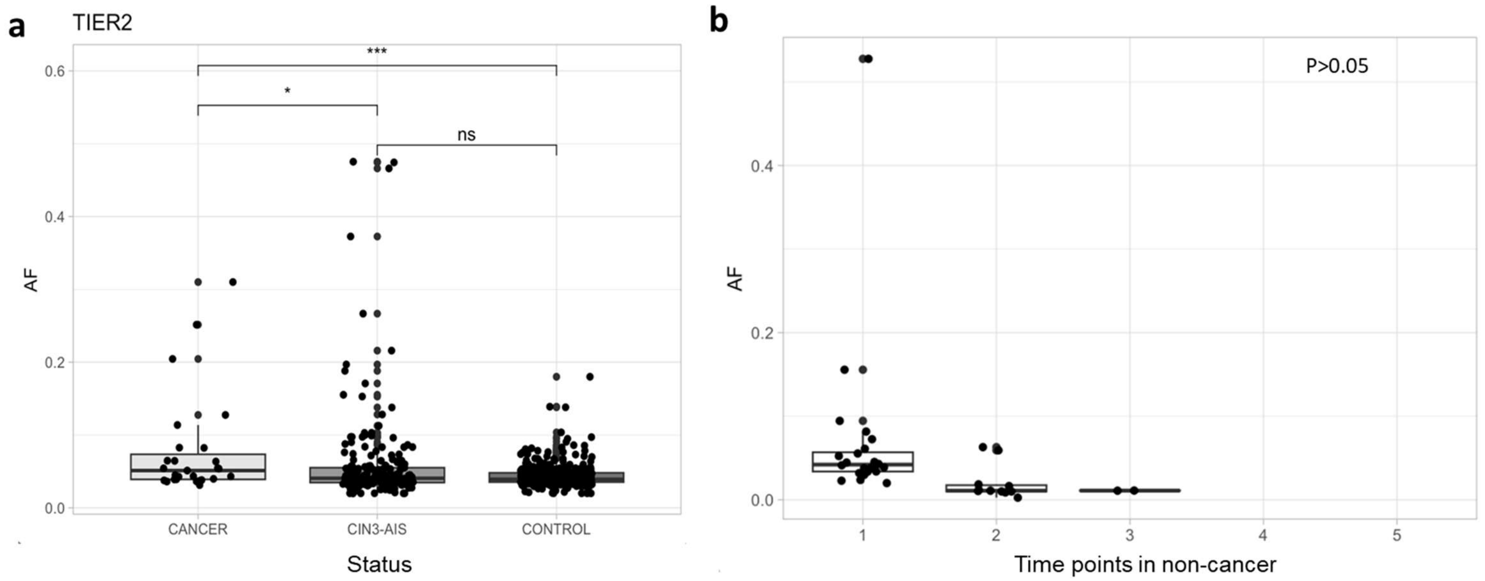

Supplementary Figure 5. Variant allele fraction (AF) of individual hotspot mutations and the AF change in multiple serial time-points. Red dashed lines = AF threshold of 0.02. Colored lines represent a woman, and dots represent samples with mutations collected from the same woman.

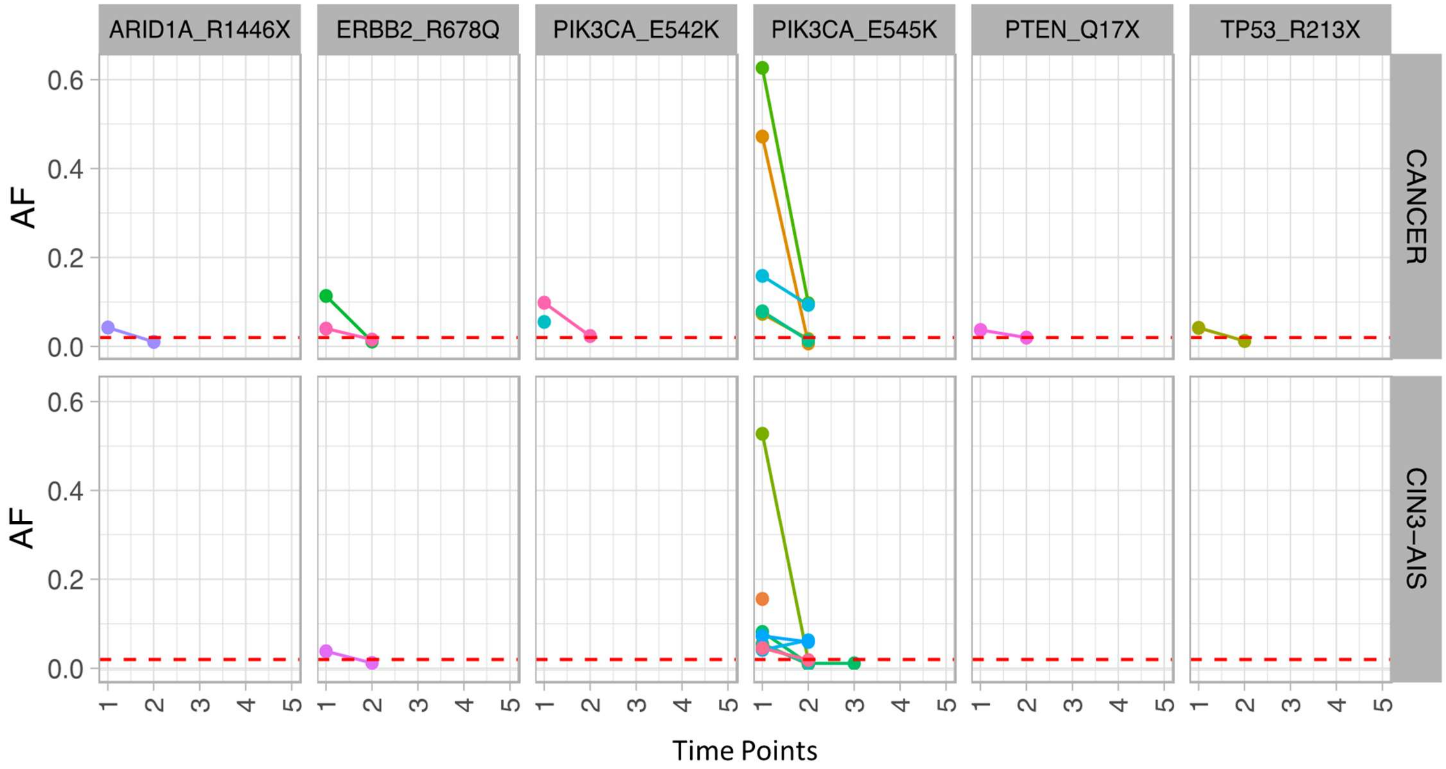

Supplementary Tables

Supplementary Table 1: Samples counts used in the "single time-point" analyses (N=3,351) by years from outcome ascertainment.

Status	Total women	Total samples	
	N	≤2y from outcome ascertainment	≥3y (max year) from outcome ascertainment
ADC	76	70	6 (5)
SCC	74	63	11 (7)
ICC unk.	11	11	0
AIS	166	157	9 (7)
CIN3	984	909	75 (10)
AIS/CIN3 unk.	1	1	0
CIN2	561	520	41 (9)
Control	1478	1300	178 (11)
**Total**	**3351**	**3031**	**320**

ADC = adenocarcinoma; SCC = squamous cell carcinoma; ICC = invasive cervical cancer; AIS = adenocarcinoma in situ; CIN3 = cervical intraepithelial neoplasia grade 3; CIN2 = CIN grade 2; unk = unknown histology. Controls include women with normal cytology/histology and low-grade lesions (ASCUS, LSIL, CIN1).

Supplementary Table 2: Number of women with a single HPV16, HPV18, or HPV45 infection and number with HR-HPV co-infections.

**Status**	HPV16 single	HPV18 single	HPV45 single	HPV16, 18	HPV16, 45	HPV16, 18, 45	HPV18, 45	HPV16, other† HR-HPV	HPV18, other† HR-HPV	HPV45, other† HR-HPV	Total women
ADC	43	21	8					2	2		76
SCC	51	8	4	1	2			8			74
ICC unk.	10	1									11
AIS	74	40	5	10	1	2	3	21	8	2	166
CIN3	612	49	27	35	20	3	4	193	28	13	984
AIS/CIN3 unk.		1									1
CIN2	159	90	51	41	28	2	10	104	46	30	561
Control	496	223	232	49	33	2	4	281	80	78	1478
**total**	**1445**	**433**	**327**	**136**	**84**	**9**	**21**	**609**	**164**	**123**	**3351**

ADC = adenocarcinoma; SCC = squamous cell carcinoma; ICC = invasive cervical cancer; AIS = adenocarcinoma in situ; CIN3 = cervical intraepithelial neoplasia grade 3; CIN2 = CIN grade 2; unk = unknown histology; † other HR-HPV indicates a co-infection with a HR-HPV type other than HPV16, HPV18 or HPV45.

Supplementary Table 3: Sample counts used in the "serial time-point" analyses (N=974) by order of collection and years from outcome ascertainment.

**Total women**		**Total samples, most recent collection***		**Total samples, prior collections**						
**Time points (TP)†**		**TP1**		**TP2**		**TP3**		**TP4**		**TP5**
**Status**	**N**	**≤2y**	**≥3y (max year)**	**≤2y**	**≥3y (max year)**	**≤2y**	**≥3y (max year)**	**≤2y**	**≥3y (max year)**	**≥3y (max year)**
Cancer	43	37	6 (8)	25	18 (9)	1	9 (8)	0	5 (9)	2 (10)
CIN3/AIS	216	200	16 (8)	138	78 (8)	11	78 (8)	0	33 (9)	3 (5)
CIN2	62	57	5 (7)	43	19 (9)	4	7 (5)	0	3 (5)	0 (-)
Control	75	59	16 (10)	63	12 (11)	10	10 (4)	3	2 (4)	1 (7)
**Total**	**396**	**353**	**43**	**269**	**127**	**26**	**104**	**3**	**43**	**6**

*also used in the cross-sectional analyses. † time-points are categorized based on the delta values between the sequential time-points and categorized based on this time-frame as ≤2years or ≥3years from diagnosis or the preceding serial sample. CIN3 = cervical intraepithelial neoplasia grade 3; AIS = adenocarcinoma in situ; CIN2 = CIN grade 2.

Supplementary Table 4: Variant allele fraction (VAF) of known target mutations called in the Acrometrix Oncology Hotspot (AOH) control samples using our panel sequencing assay.

Control ID	VAF range	Total mutations called	N, mutations kept after filters	% of mutations kept after filters
AOH-T1	(0.02,0.05]	8	6	75.00%
	(0.05,0.15]	87	86	98.90%
	(0.15,0.35]	21	18	85.70%
	(0.35,1]	5	5	100.00%
	**Total**	121	115	77.70%
AOH-T2	(0.02,0.05]	10	7	70.00%
	(0.05,0.15]	86	84	97.70%
	(0.15,0.35]	20	18	90.00%
	(0.35,1]	6	6	100.00%
	**Total**	122	115	77.70%
AOH-T3	(0.02,0.05]	6	5	83.30%
	(0.05,0.15]	93	91	97.80%
	(0.15,0.35]	17	15	88.20%
	(0.35,1]	6	6	100.00%
	**Total**	122	117	79.10%

Supplementary Table 5: Association of TIER2 hotspot mutations with precancers and cancers among samples collected within 2 years of outcome ascertainment.

**Status**		**no HS mutation**		**≥1 HS mutations**					
	**Total**	**N**	**%**	**N**	**%**	**P**	**OR**	**95%CI**	
**Control**	1300	1114	85.7%	186	14.3%	*ref*			
**CIN2**	520	461	88.7%	59	11.3%	0.10	0.77	0.56	1.05
**CIN3/AIS**	1067	952	89.2%	115	10.8%	**0.01**	0.72	0.56	0.93
**CIN3**	909	820	90.2%	89	9.8%	**$1.7x10^{-3}$**	0.65	0.50	0.85
**AIS**	157	131	83.4%	26	16.6%	0.45	1.19	0.76	1.86
**Cancer**	144	122	84.7%	22	15.3%	0.75	1.08	0.67	1.75
**SCC**	63	57	90.5%	6	9.5%	0.29	0.63	0.27	1.48
**ADC**	70	57	81.4%	13	18.6%	0.33	1.37	0.73	2.54
		**N**	**%**	**N, *PIK3CA***	**%**				
**Control**	1118	1114	99.6%	4	0.4%	*ref*			
**Cancer**	122	122	100.0%	0	0.0%	0.765	-	-	-
		**N**	**%**	**N, non-*PIK3CA***	**%**				
**Control**	1285	1114	86.7%	171	13.3%	*ref*			
**Cancer**	142	122	85.9%	20	14.1%	0.79	1.07	0.65	1.76
**SCC**	63	57	90.5%	6	9.5%	0.39	0.69	0.29	1.61
**ADC**	68	57	83.8%	11	16.2%	0.50	1.26	0.65	2.45

CIN2 = cervical intraepithelial neoplasia grade 2; CIN3 = CIN grade 3, AIS = adenocarcinoma in situ; SCC = squamous cell carcinoma, ADC = adenocarcinoma; HS = hotspot; P = P value by multinomial logistic regression; OR = odds ratio; CI = confidence interval. OR, 95%CI, and P values were estimated using multinomial logistic regression; tests were two-sided. Significant P values are bolded.

Supplementary Table 6: Distribution of hotspot mutations identified in cancers compared with their prevalence in controls.

TIER			No HS mutation		≥1 HS mutations		
	**Status**	**Total**	**N**	**%**	**N**	**%**	**P**
**TIER1**	Control	1300	1277	98.2%	23	1.8%	
	Cancer	144	107	74.3%	37	25.7%	**$<2.2 \times 10^{-16}$**
**TIER2**	Control	1300	1248	96.0%	52	4.0%	
	Cancer	144	122	84.7%	22	15.3%	**8.2×10^{-7}**

HS = hotspot. P values estimated with two-sided Fisher's exact tests. Significant P values are bolded.

Supplementary Table 7: Associations of hotspot mutations matching APOBEC3 and non-APOBEC3 motifs for TIER1 and by HPV type.

HPV	Status	Total	No HS mutation		≥1 HS mutations		P	OR	95%CI	
**HPV16**			**N**	**%**	**N, APOBEC3**	**%**				
	**Control**	648	645	99.5%	3	0.5%	*ref*			
	**Cancer**	92	75	81.5%	17	18.5%	**$1.1x10^{-9}$**	48.7	14.0	170.2
			**N**	**%**	**N, non-APOBEC3**	**%**				
	**Control**	659	645	97.9%	14	2.1%	*ref*			
	**Cancer**	84	75	89.3%	9	10.7%	**$1.2x10^{-4}$**	5.5	2.3	13.2
**HPV18/45**			**N**	**%**	**N, APOBEC3**	**%**				
	**Control**	548	543	99.1%	5	0.9%	*ref*			
	**Cancer**	34	30	88.2%	4	11.8%	**$1.2x10^{-4}$**	14.5	3.7	56.7
			**N**	**%**	**N, non-APOBEC3**	**%**				
	**Control**	552	543	98.4%	9	1.6%	*ref*			
	**Cancer**	34	30	88.2%	4	11.8%	**$9.3x10^{-4}$**	8.0	2.3	27.6

Samples with co-occurrence of both APOBEC3 and non-APOBEC3 induced mutations were excluded from the analyses. P = logistic regression; HS = hotspot; OR = odds ratio; CI = confidence interval. Significant P values are bolded. OR, 95%CI, and P values were estimated using logistic regression; tests were two-sided

Supplementary Table 8: Association of hotspot mutations with precancers and cancers among samples collected ≥3 years from outcome ascertainment.

TIER	Status		no HS mutation		≥1 HS mutations		P	OR	95%CI	
		Total	N	%	N	%				
TIER1	Control	178	173	97.2%	5	2.8%				
	CIN2	41	41	100.0%	0	0.0%	0.93	-	-	-
	CIN3/AIS	84	81	96.4%	3	3.6%	0.74	1.28	0.30	5.49
	CIN3	75	73	97.3%	2	2.7%	0.95	0.95	0.18	5.00
	AIS	9	8	88.9%	1	11.1%	0.20	4.33	0.45	41.47
	Cancer	17	15	88.2%	2	11.8%	0.08	4.61	0.82	25.82
	SCC	11	9	81.8%	2	18.2%	**0.02**	7.68	1.31	45.17
	ADC	6	6	100.0%	0	0.0%	0.91	-	-	-
TIER2	Control	178	163	91.6%	15	8.4%				
	CIN2	41	37	90.2%	4	9.8%	0.79	1.17	0.37	3.74
	CIN3/AIS	84	76	90.5%	8	9.5%	0.77	1.14	0.47	2.81
	CIN3	75	68	90.7%	7	9.3%	0.82	1.12	0.44	2.87
	AIS	9	8	88.9%	1	11.1%	0.78	1.36	0.16	11.61
	Cancer	17	14	82.4%	3	17.6%	0.22	2.33	0.60	9.02
	SCC	11	10	90.9%	1	9.1%	0.94	1.09	0.13	9.08
	ADC	6	4	66.7%	2	33.3%	0.06	5.43	0.92	32.15

HS = hotspot; OR = odds ratio; CI = confidence interval; P=multinomial logistic regression. Significant P values are bolded. OR, 95%CI, and P values were estimated using multinomial logistic regression; tests were two-sided.

Supplementary Table 9: Samples included in time-point 1 (TP1) with hotspot mutations.

Status	All			≤2 years			≥3 years		
	Total	samples with ≥1 TIER1/2 HS mutations		Total	samples with ≥1 TIER1/2 HS mutations		Total	samples with ≥1 TIER1/2 HS mutations	
	N	N	%	N	N	%	N	N	%
Cancer	43	15	34.9%	37	13	35.1%	6	2	33.3%
CIN3/AIS	216	15	6.9%	200	15	7.5%	16	0	0.0%
CIN2	62	2	3.2%	57	2	3.5%	5	0	0.0%
Control	75	3	4.0%	59	3	5.1%	16	0	0.0%
**Total**	396	35	8.8%	353	33	9.3%	43	2	4.7%

CIN2 = cervical intraepithelial neoplasia grade 2; CIN3 = CIN grade 3, AIS = adenocarcinoma in situ; HS = hotspot.
