# Supplementary material for: Somatic mutations in 3929 HPV positive cervical cells associated with infection outcome and HPV type
Source: Nat Commun. 2024 Sep 12;15:7895. doi: 10.1038/s41467-024-51713-y (PMC11393421; doi:10.1038/s41467-024-51713-y)
Supplement: Supplementary file 3 — Description of Additional Supplementary Files [file 41467_2024_51713_MOESM3_ESM.pdf]

Description of Additional Supplementary Files

File Name: Supplementary Data 1

Description: Individual sample coverage metrics across the 20 genes targeted by our panel for all 3,929 single time-point and serial samples.

File Name: Supplementary Data 2

Description: List of the 3,192 mutated sites evaluated and their TIER classification.

Legend: REF = reference allele; ALT = alternative allele; c.DNA = DNA sequence change; Alteration = amino acid change for the nonsynonymous mutations.
