# Supplementary material for: Somatic mutations in 3929 HPV positive cervical cells associated with infection outcome and HPV type
Source: Nat Commun. 2024 Sep 12;15:7895. doi: 10.1038/s41467-024-51713-y (PMC11393421; doi:10.1038/s41467-024-51713-y)
Supplement: Supplementary file 6 — Reporting Summary [file 41467_2024_51713_MOESM6_ESM.pdf]

nature portfolio

Corresponding author(s): Lisa Mirabello

Last updated by author(s): Jul 9, 2024

Reporting Summary

Nature Portfolio wishes to improve the reproducibility of the work that we publish. This form provides structure for consistency and transparency in reporting. For further information on Nature Portfolio policies, see our Editorial Policies and the Editorial Policy Checklist.

Statistics

For all statistical analyses, confirm that the following items are present in the figure legend, table legend, main text, or Methods section.

n/a	Confirmed	
☐	☒	The exact sample size (*n*) for each experimental group/condition, given as a discrete number and unit of measurement
☐	☒	A statement on whether measurements were taken from distinct samples or whether the same sample was measured repeatedly
☐	☒	The statistical test(s) used AND whether they are one- or two-sided<br>*Only common tests should be described solely by name; describe more complex techniques in the Methods section.*
☐	☒	A description of all covariates tested
☐	☒	A description of any assumptions or corrections, such as tests of normality and adjustment for multiple comparisons
☐	☒	A full description of the statistical parameters including central tendency (e.g. means) or other basic estimates (e.g. regression coefficient) AND variation (e.g. standard deviation) or associated estimates of uncertainty (e.g. confidence intervals)
☐	☒	For null hypothesis testing, the test statistic (e.g. *F*, *t*, *r*) with confidence intervals, effect sizes, degrees of freedom and *P* value noted<br>*Give P values as exact values whenever suitable.*
☒	☐	For Bayesian analysis, information on the choice of priors and Markov chain Monte Carlo settings
☒	☐	For hierarchical and complex designs, identification of the appropriate level for tests and full reporting of outcomes
☒	☐	Estimates of effect sizes (e.g. Cohen's *d*, Pearson's *r*), indicating how they were calculated

Our web collection on statistics for biologists contains articles on many of the points above.

Software and code

Policy information about availability of computer code

Data collection	No software was used for data collection.
Data analysis	We used Torrent Suite software (Thermo Fisher Scientific, Waltham, MA, USA) for sequence read quality assessment and trimming, and mapping to the human reference genome. Single nucleotide variants were called using the Torrent Variant Caller (TVC) v.5.0.3 (Thermo Fisher Scientific, Waltham, MA, USA). SnpEff v.3.6c was used to annotate synonymous and nonsynonymous mutations, indels, and frameshifts, and Annovar software (Wang et al., 2010) was used to annotate exonic and non-exonic mutations, the gnomAD frequencies, and COSMIC v92 information. We used the following somatic mutation databases to identify somatic hotspot mutations previously described: the Cancer Genome Interpreter (CGI) (Tamborero et al., 2018), Mutagene (Goncearenco et al., 2017; Brown et al., 2019), cBioPortal-TCGA-cervix (Cervical Squamous Cell Carcinoma and Endocervical Adenocarcinomas) (Cerami et al., 2012; Gao et al., 2013), and CHASMplus (Tokheim & Karchin, 2019). For HPV16/18/45 sequences, type-specific lineage/sublineage assignment was based on a maximum likelihood phylogenetic tree created with RAxML MPI (Stamatakis, 2006). All statistical analyses were performed in R version 4.1.0.

For manuscripts utilizing custom algorithms or software that are central to the research but not yet described in published literature, software must be made available to editors and reviewers. We strongly encourage code deposition in a community repository (e.g. GitHub). See the Nature Portfolio guidelines for submitting code & software for further information.

Data

Policy information about availability of data

All manuscripts must include a data availability statement. This statement should provide the following information, where applicable:

- Accession codes, unique identifiers, or web links for publicly available datasets
- A description of any restrictions on data availability
- For clinical datasets or third party data, please ensure that the statement adheres to our policy

Somatic data for the 20 genes, and de-identified phenotype information, used in our study is publicly available with accession numbers phs003691

Research involving human participants, their data, or biological material

Policy information about studies with human participants or human data. See also policy information about sex, gender (identity/presentation), and sexual orientation and race, ethnicity and racism.

Reporting on sex and gender	Our study included only females.
Reporting on race, ethnicity, or other socially relevant groupings	Our study included self-reported race and ethnicity information from electronic health records. Participants self-reported as White, Hispanic, Asian/Pacific Islander, Black, Multi-racial, or other.
Population characteristics	A total of 3,351 women were included in our study: 1,478 controls, 561 cervical intraepithelial neoplasia grade 2 (CIN2; equivocal squamous precancer), 984 CIN grade 3 (CIN3; squamous precancer), 166 adenocarcinoma in situ (glandular precancer), 1 precancer with unknown histology, 74 squamous cell carcinoma, 76 adenocarcinoma, and 11 invasive cervical cancer with unknown histology. Controls were defined as women having baseline HPV16-, HPV18- and/or HPV45-positive samples that subsequently cleared their infection and/or had an infection defined as normal or low-grade lesion with no histologic evidence of equivocal precancer or worse (CIN2+) during the study follow-up period. The average age of the women included in our study was 38 years (SD 11), and the majority self-reported their race/ethnicity as White (51%), followed by Hispanic (20%), Asian/PI (15%), Black (8%), or multiracial/other (7%).
Recruitment	Residual exfoliated cervical cell samples for our study were selected from women in the Kaiser Permanente Northern California (KPNC) – National Cancer Institute HPV Persistence and Progression (PaP) cohort. The PaP cohort includes approximately 55,000 women out of approximately 1 million who underwent routine cervical cancer screening between December 2006 and January 2011 at KPNC. Participants could opt-out of retaining residual cervical specimens from pap-smears and those samples were discarded (~8% of women opted out). Women were followed over time and we obtained coded information on subsequent cervical cancer screening test results and histology results from electronic health records through 2019. For our study, we selected cervical cell samples from women positive for HPV16, HPV18 and/or HPV45, including all available precancer/cancer cases and approximately 1 control per case randomly selected for comparison.
Ethics oversight	The KPNC institutional review board (IRB) approved use of the data, and the National Institutes of Health Office of Human Subjects Research deemed this study exempt from IRB review.

Note that full information on the approval of the study protocol must also be provided in the manuscript.

Field-specific reporting

Please select the one below that is the best fit for your research. If you are not sure, read the appropriate sections before making your selection.

☒ Life sciences ☐ Behavioural & social sciences ☐ Ecological, evolutionary & environmental sciences

For a reference copy of the document with all sections, see nature.com/documents/nr-reporting-summary-flat.pdf

Life sciences study design

All studies must disclose on these points even when the disclosure is negative.

Sample size	A total of 3,351 women were included in our study: 1,478 controls, 561 cervical intraepithelial neoplasia grade 2 (CIN2; equivocal squamous precancer), 984 CIN grade 3 (CIN3; squamous precancer), 166 adenocarcinoma in situ (AIS; glandular precancer), 1 precancer with unknown histology, 74 squamous cell carcinoma (SCC), 76 adenocarcinoma (ADC), and 11 invasive cervical cancer (ICC) with unknown histology. We selected all available HPV16-, HPV18- and HPV45-positive precancer (CIN3, AIS) and cancer (ICC, SCC, ADC) samples. We included all available precancer and cancer cases since these are rare outcomes of HPV infection, and then randomly selected approximately one control per CIN3/AIS/cancer case. In addition, 396 women in our study had at least one additional serial sample, collected prior to their most recent screening visit (N=974 samples), available for inclusion (serial time-point samples). We included all available serial samples. In total, there were 3,929 samples collected from 3,351 women in the study.
Data exclusions	We included only included samples from women that were HPV16-, HPV18- and/or HPV45-positive with the infection outcomes as noted.
Replication	To assess the performance of our assay to detect known somatic mutations at a low VAF and to establish filters to clean potential false

Replication	positive variants, we sequenced the Acrometrix Oncology Hotspot Control DNA (AOH) (Thermo Fisher Scientific, Waltham, MA, USA). This control panel consists of a mixture of genomic DNA, derived from the cell line DM24385 used to develop the Genome in a Bottle reference genome (Zook et al., 2016), and synthetic DNA, with known somatic mutations in cancers introduced at low variant allelic fractions. We sequenced this AOH DNA in triplicate, as part of 3 different experimental plates. We evaluated the expected and observed mutations to optimize detection of somatic mutations.<br>For each sample plate, we included a water blank and a positive human control sample (HeLa, SiHa), plus 5 sample replicates.
Randomization	We included all available precancer and cancer cases, and randomly selected approximately one control per CIN3/AIS precancer or cancer case.
Blinding	The laboratory performing the sequencing assays were blinded to the infection outcome status.

Reporting for specific materials, systems and methods

We require information from authors about some types of materials, experimental systems and methods used in many studies. Here, indicate whether each material, system or method listed is relevant to your study. If you are not sure if a list item applies to your research, read the appropriate section before selecting a response.

Materials & experimental systems

n/a	Involved in the study
☒	☐ Antibodies
☒	☐ Eukaryotic cell lines
☒	☐ Palaeontology and archaeology
☒	☐ Animals and other organisms
☒	☐ Clinical data
☒	☐ Dual use research of concern
☒	☐ Plants

Methods

n/a	Involved in the study
☒	☐ ChIP-seq
☒	☐ Flow cytometry
☒	☐ MRI-based neuroimaging

Plants

Seed stocks	*Report on the source of all seed stocks or other plant material used. If applicable, state the seed stock centre and catalogue number. If plant specimens were collected from the field, describe the collection location, date and sampling procedures.*
Novel plant genotypes	*Describe the methods by which all novel plant genotypes were produced. This includes those generated by transgenic approaches, gene editing, chemical/radiation-based mutagenesis and hybridization. For transgenic lines, describe the transformation method, the number of independent lines analyzed and the generation upon which experiments were performed. For gene-edited lines, describe the editor used, the endogenous sequence targeted for editing, the targeting guide RNA sequence (if applicable) and how the editor was applied.*
Authentication	*Describe any authentication procedures for each seed stock used or novel genotype generated. Describe any experiments used to assess the effect of a mutation and, where applicable, how potential secondary effects (e.g. second site T-DNA insertions, mosiacism, off-target gene editing) were examined.*
